# Correction: Where to Go: Breaking the Symmetry in Cell Motility

**DOI:** 10.1371/journal.pbio.1002510

**Published:** 2016-06-22

**Authors:** The PLOS Biology Staff

## Notice of Republication

This article was republished on June 10, 2016, to correct errors in the references that were introduced during the typesetting process. Please download this article again to view the correct version. The originally published, uncorrected article and the republished, corrected article are provided here for reference.

## Supporting Information

S1 File Originally published, uncorrected article. (PDF)

S2 File Republished corrected article. (PDF)
