# Supplementary material for: Correction: Where to Go: Breaking the Symmetry in Cell Motility
Source: PLoS Biol. 2016 Jun 22;14(6):e1002510. doi: 10.1371/journal.pbio.1002510 (PMC4917171; doi:10.1371/journal.pbio.1002510)
Supplement: S1 File — (PDF) [file pbio.1002510.s001.PDF]

PLOS | BIOLOGY

PRIMER

Where to Go: Breaking the Symmetry in Cell Motility

Sui Huang*

Institute for Systems Biology, Seattle, Washington

* sui.huang@systemsbiology.org

Abstract

Cell migration in the "correct" direction is pivotal for many biological processes. Although most work is devoted to its molecular mechanisms, the cell's preference for one direction over others, thus overcoming intrinsic random motility, epitomizes a profound principle that underlies all complex systems: the choice of one axis, in structure or motion, from a uniform or symmetric set of options. Explaining directional motility by an external chemo-attractant gradient does not solve but only shifts the problem of causation: whence the gradient? A new study in PLOS Biology shows cell migration in a self-generated gradient, offering an opportunity to take a broader look at the old dualism of extrinsic instruction versus intrinsic symmetry-breaking in cell biology.

CrossMark
click for updates

OPEN ACCESS

Citation: Huang S (2016) Where to Go: Breaking the Symmetry in Cell Motility. PLoS Biol 14(5): e1002463. doi:10.1371/journal.pbio.1002463

Published: May 19, 2016

Copyright: © 2016 Sui Huang. This is an open access article distributed under the terms of the Creative Commons Attribution License, which permits unrestricted use, distribution, and reproduction in any medium, provided the original author and source are credited.

Funding: The author's work is supported by the National Health Institutes (5R01GM109964-02) and the Institute for Systems Biology. The funders had no role in study design, data collection and analysis, decision to publish, or preparation of the manuscript.

Competing Interests: The author has declared that no competing interests exist.

When you come to a fork in the road, take it.–*Yogi Berra 1925–2015*

Introduction

Cell locomotion, driven by momentum generated from the cell, is considered a major invention in evolution [1,2], or even a fundamental cell functionality itself [3]. Cells capable of locomotion do not rely on diffusion or convection in the outside world, such as winds or currents, to find nutrients or partners for sexual reproduction. Primitive locomotion consists of uncontrolled jumps in any direction, which results in random walk: an erratic walk in which direction changes randomly after every step [4]. The capacity of such apparently aimless migration offered an advantage over sessile lifeforms, however, by allowing a larger physical space to be explored in a static environment. The next major step in evolution involved harnessing the random walk to produce motility in the desired direction, an innovation that evolved in all three domains of life: Bacteria, Archaea, and Eukarya.

Investigations in cell motility have largely focused on its molecular underpinning, such as pili in bacteria [5] or the cytoskeleton of lamellipodia in eukaryotic cells [6,7], as well as the pathways that mediate chemotactic sensing and send directional cues to the locomotion apparatus. What has received less attention, but is nonetheless a profound problem in the evolution of complexity, is not the machinery that allows motility but the principles that govern directionality.

What drives directed motility? When a single-cell microorganism migrates toward a food source, the "cause" of its directed movement appears obvious [8]: the food molecules, e.g.,

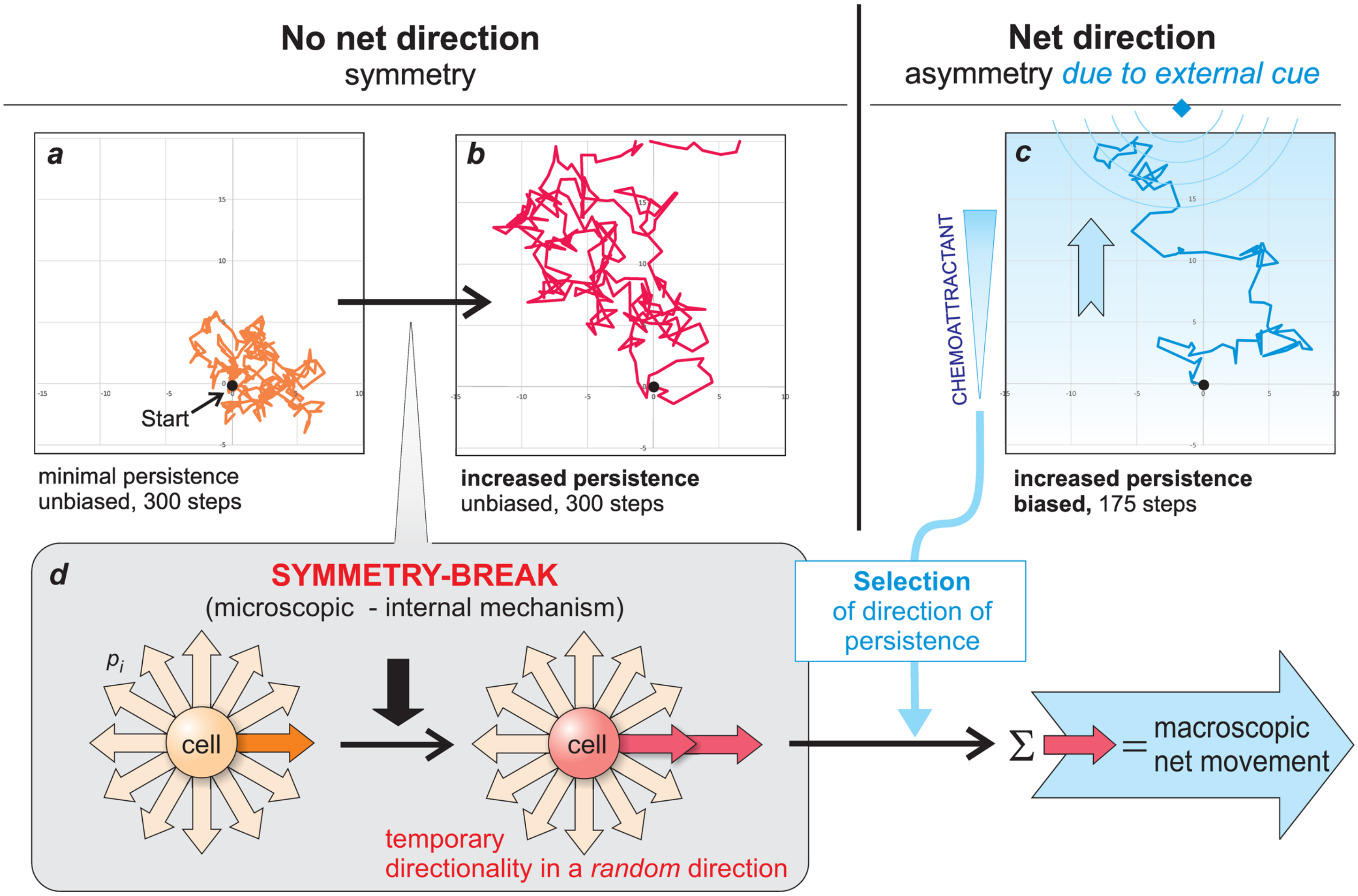

Fig 1. Symmetry-breaking at lower level in directional motility: overcoming random walk. Computer-simulated trace of random walk with minimal persistence (a), increase persistence (probabilistically increased step length) (b), and biased random walk (c) based on the former (b), which was subjected to directional bias by a gradient from a chemoattractant source on the top of the field. Note the short, net distance travelled to the source at the top after only 175 steps in (c), whereas in undirected persistent walk (b), only after 300 steps does the spread temporarily cover the location of the source. Microscopic symmetry-breaking event (d) creates a temporary directionality of the random steps to increase persistence. This symmetric break is the basis for the macroscopic directionality instructed by an external gradient, which by definition is not a symmetry-break.

doi:10.1371/journal.pbio.1002463.g001

glucose, diffuse and establish a chemotactic gradient that serves as a directional guide. Similarly, in multicellular organisms, cells migrate, guided by morphogens and chemokines, to the places where they are required to form tissue patterns, defend against microbial invaders, or restore tissue integrity.

The most primitive form of motility is random walk, manifest in the wiggling trace of a migrating cell that is equivalent to that of a single molecule in Brownian movement (Fig 1A) [4,9]: the cell does not maintain a steady direction. Instead, if for argument's sake we view locomotion as a series of discrete steps, it moves in an arbitrary direction each time it makes a step. Because of this lack of a characteristic direction, random walk is "symmetric." Directionality is then in essence the overcoming of random walk by biasing the probabilistic steps toward the same direction as the last one, such that the cell moves for a longer period of time in one direction. The latter is characterized by a key parameter of random walk, the persistence time (or length) [10]. Increasing persistence is achieved by temporary suppression of the random turning at each step of locomotion. It enlarges the space for random search, but motility still has no

preferred overall "net" direction (Fig 1B). However, a temporary increase in persistence disrupts the symmetric randomness and locks motility in an arbitrary direction for a few steps. It is in this sense that increasing persistence is a local (microscopic but not macroscopic) "breaking" of the symmetry. This will be important later.

This Primer will discuss the phenomenon of directionality within the wider context of the elementary principle of symmetry-breaking [11–13] and will not consider the marvelous molecular machinery that implements directed motility or chemotaxis. Symmetry-breaking offers a general framework for comparing various types of motility, puts molecular mechanisms in a new context, and provides an alternative perspective for our quest to comprehend the evolution of complex patterns and to link them to physicochemical reality.

Symmetry-Breaking

A central question is whether the "direction" in directional motility is instructed by an external clue or is the result of internal decision making—akin to the unfortunate person lost in a desert who decides to follow the old wisdom of walking straight in one (any) direction to avoid wandering in a circle. Avoiding a circle—the shape with perfect, hence highest, symmetry—is an example of a symmetry-breaking event in the technical sense (see Box 1 and Fig 2) [13].

First what does "breaking the symmetry" mean? A more general qualitative definition of symmetry-breaking in a system will suffice here: it is the spontaneous emergence from within a system (under a defined condition) of new, discrete structural or behavioral options in a previously indifferent, mostly continuous space of options. The water wheel in Fig 2A provides a toy example. The conversion of the potential well to a hilltop, pushing the marble in it to one of two possible slopes of the hill, representing a so-called symmetry-breaking bifurcation, is a more formal example (Fig 2B). The key idea is that once symmetry is broken, the system must make a choice, which is random (hence intrinsic) if external bias is absent.

In biology, a prosaic example of symmetry-breaking is the generation of the animal-vegetative polarity in a perfectly, hence maximally, symmetric spherical egg (Fig 3): with the polarization of the egg, the infinite symmetry is reduced by the acquisition of one symmetry axis, suddenly creating the question as to how the cell lies with respect to the previously inexistent axis of symmetry. Since at this point it is inevitable to think of external factors that influence the direction of the axis (gravity, extracellular matrix), it is of semasiological importance to keep in mind that symmetry-breaking arises from intrinsic properties of the system's design and is not the result of instruction by an external signal. It is a system-immanent ("built-in") capacity: under some system configuration, the system is poised to undergo a symmetry break. Thereby, new options that break the system's symmetry are generated that the system has to implement and in which it is then locked-in (at least for a while). The question of which option, e.g., rotate left or right, or move in this or that direction, is executed can then be determined by externalities: a fluctuation from noise or a specific instructive cue, such as the gravity of a chemoattractant, that biases the choice. Thus, external factors do not create the options but just tip the system poised to choose between distinct options.

In summary, the actual event of symmetry-breaking, the creation of the possibility of asymmetric behavior, is not "caused" by an external conductor that orchestrates the change in system behavior, or an external template that imposes a pattern, or a helmsman that navigates the movement in the desired direction. These apparent extrinsic causative factors only make the choice once the symmetry is poised to be broken. Thus, symmetry-breaking is said to be a spontaneous, intrinsic process.

Box 1. The Principle of Symmetry-Breaking

Symmetry-breaking can be illustrated by a toy model, a simple water wheel as shown in Fig 2A. A more formal example is the symmetry-breaking bifurcation from non-linear dynamical systems theory that deals with the stability of states that can be visualized as a landscape (Fig 2B).

In the water wheel model (Fig 2A), in the absence of water flow, there is no preferred direction of rotation (wind may rock the wheel back and forth): any angular position of the wheel is possible—the wheel is said to be in an indifferent equilibrium. But once water flows, coming exactly centered from the top, and fills the top bucket, the system is destabilized ("poised") and the wheel has to choose one direction of rotation, clockwise or counter-clockwise. Without any bias, the choice will be random, determined by minute details ("sensitivity to initial conditions"). Once it starts turning, symmetry is broken: the wheel will keep rotation in the same direction, bringing empty buckets back up to the water source, and its new weight will drive the rotation in the same direction because of the existing momentum of the rotation. Mechanistically, choice of direction of rotation is influenced by external factors, e.g., where the very first droplet falls, gentle "tipping" of the wheel into one direction by wind, etc. The result is that microscopic asymmetry is created and then translated into macroscopic asymmetry of rotating in one direction.

In the theory of nonlinear dynamical systems, a bifurcation event can result in a symmetry-breaking in that a system has to make a decision to occupy one of two distinct, newly available stable states after the existing one has been destabilized (Fig 2B). Here, a system's state *S* can be displayed as a marble on a (quasi)-potential landscape [14], with the *x*-axis (black arrow) as the state variable *S*. A classical scenario of symmetry-breaking occurs when a particular parameter of the system (the bifurcation parameter, blue arrow) gradually changes, which translates into a change in the shape of the landscape topology, such that the existing stable equilibrium state of the system (*A*, orange marble) is destabilized and converted into an unstable equilibrium ("poised state;" red marble), at which point two alternative stable states (*B*, *C*) become accessible, from among which the poised system has to choose one. This is the bifurcation event that breaks the symmetry because it creates the options, only one of which will be realized. It is here that external factors exert their influence: a minimal, often conspicuous input, such as one exerted by tiny random molecular fluctuations, will "tip" the poised system toward one of the two stable states that is then maintained for a while. The inset in Fig 2B shows the formal representation of this behavior as a bifurcation diagram in which the solid black curves represent the position of the stable equilibrium states (value of *S*). Dashed curve represents the unstable poised state. In this case the symmetry-break is effectively a "fork" that must be "taken" if the cell enters the bistable regime. This theoretical model accounts for the notion of "symmetry-breaking bifurcation" and "tipping points." Note that the symmetric states preceding symmetry-breaking can be indifferent, in the case of the water wheel (Fig 2A), or the dynamical system's stable equilibrium state A (Fig 2B).

The Dualism of Intrinsic versus Extrinsic Causation of Patterns

To illustrate a historical debate on the dualism between intrinsic symmetry-breaking and external instruction, let us more broadly discuss pattern formation in ontogenesis: How do patterns, such as spots and stripes on animals (Fig 3D), repeated elements on body plans or digits, arise

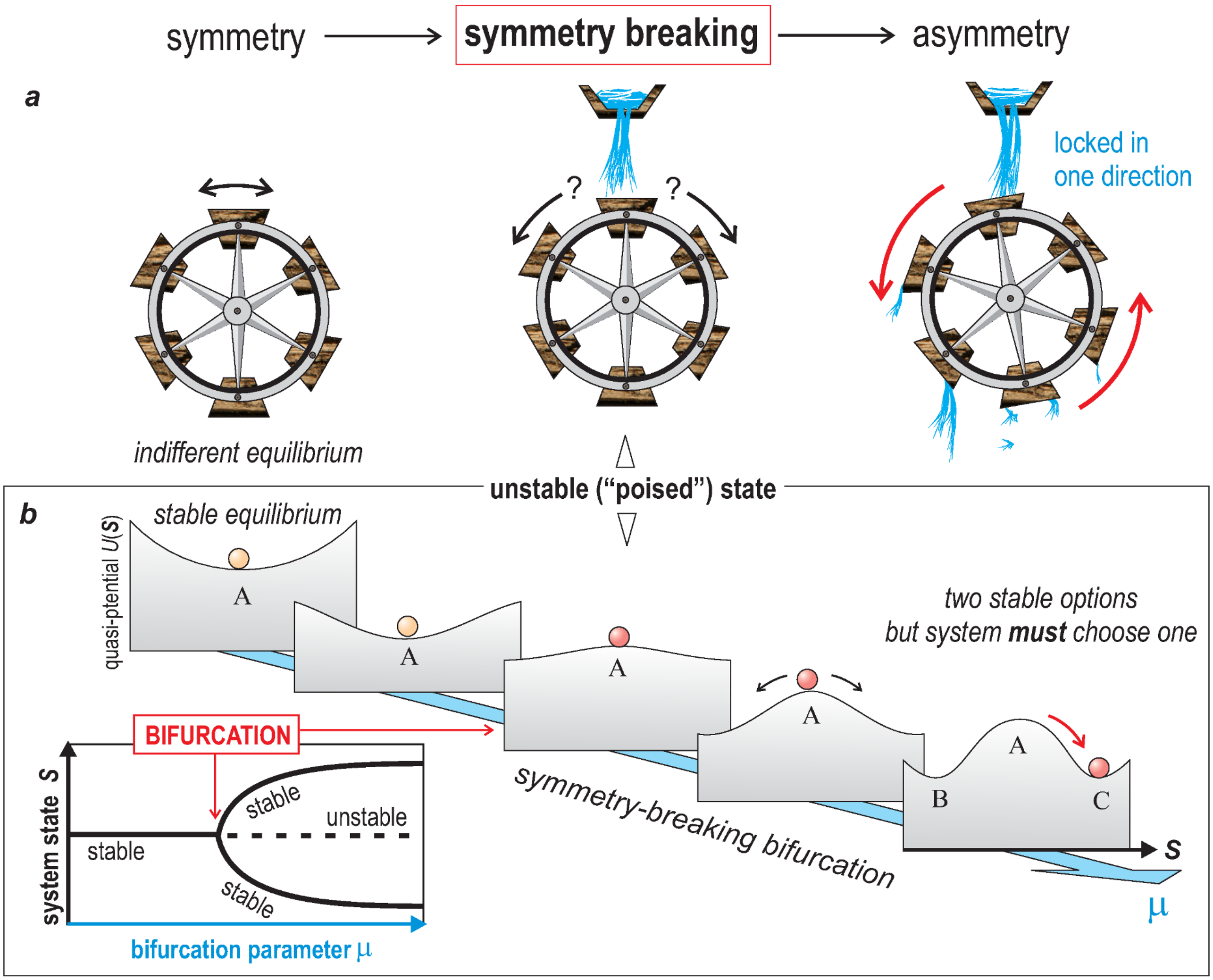

Fig 2. Symmetry-breaking. Schematic example that illustrate the concept of symmetry-breaking as explained in Box 1. ***a***. The waterwheel toy model that provides an intuitive notion of a mechanical symmetry-breaking. ***b***. The non-linear dynamics concept of a "pitch fork" bifurcation that breaks the symmetry of stable state A and its representation as quasi-potential landscape (for details see Box 1).

doi:10.1371/journal.pbio.1002463.g002

from the indifferent uniformity of the embryonic tissue? Are these patterns truly the result of intrinsic symmetry-breaking [11], or are they simply imposed by an external template or guide, without spontaneous symmetry-breaking? These alternative explanations are rooted in distinct epistemic cultures of thought in science.

In 1952, Alan Turing famously proposed that pattern formation in embryos results from the interplay of a locally activated and long-range inhibitory diffusing morphogens [16]. Formulated as partial differential equations, certain conditions (parameters, boundary conditions) can create, without external template, patterns in an initially homogeneous tissue: stripes, spots, traveling spiral waves, etc. (see [16,17] for review). At the core of the mathematical solution are symmetry-breaking bifurcations [18], and the information for the patterns is inherent in the nature of the rules that govern the behaviors of the morphogens that were initially uniformly distributed. Some Turing patterns, readily demonstrated in computer simulations in a variety of versions, have been experimentally validated (Fig 3C and 3D). Based on the outcomes of specific molecular manipulations that were predicted by theory, Turing-like

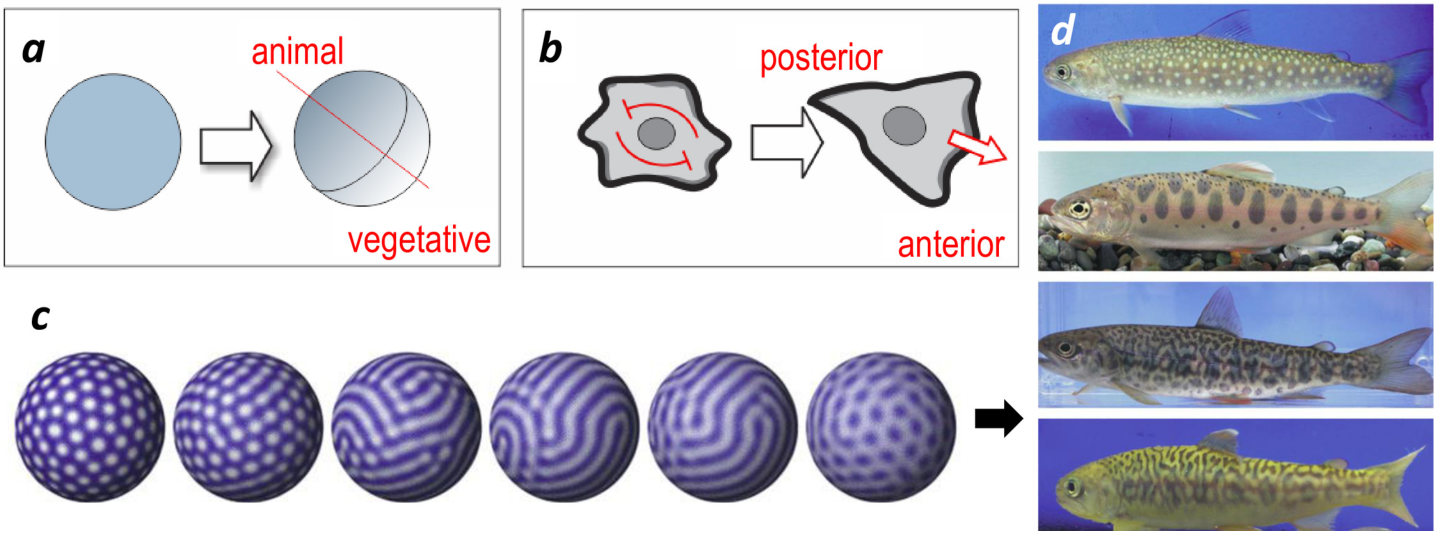

Fig 3. Examples of symmetry-breaking. ***a***. Formation of polarity in zygotes introduces a rotational symmetry axis from the animal to the vegetative pole that are distinct, breaking the symmetry of a sphere. ***b***. Polarity of a migrating metazoan cell on a planar surface (schematic). Underlying symmetry-breaking is believed to result from the mutual inhibition between cytoskeletal structures at the prospective anterior and posterior front, allowing chemo-attracting factors to reinforce and define the direction of the axis. ***c***. Computer simulations of Turing (reaction-diffusion) pattern hybridization, explaining the various patterns in hybrids of salmonid fishes [15]. The images used in ***d*** are sourced from [15], and permission has been granted to publish them here under a Creative Commons Attribution license.

doi:10.1371/journal.pbio.1002463.g003

mechanisms have been implicated in digit formation in mice, stripe patterns in fish coats, and in other repetitive patterns [17,19,20]. Moreover, specific secreted molecules, such as Nodal, FGF, BMP, and Wnt, could be shown to play the role of Turing's morphogens [21–23]. In the domain of theoretical modeling, Turing equations offered a versatile general principle that could be extended to include more components than one inhibitor and one activator, and they were linked to the bifurcation theory as the latter consolidated in the 1970s (Box 1, Fig 2B) [18]. The class of Turing models has also been used to explain cell polarization (Fig 3B) and formation of growth cones that provide directional sensitivity for chemotaxis in *Dictyostelium* [24].

By contrast, other patterns that intuit a Turing mechanism, such as spatial patterns of gene expression in the *Drosophila* embryo, turned out to be readily explained by extrinsic instruction, a pre-laid asymmetry in the localization of maternal mRNAs [25,26]. Thus, details of the actual physicochemical mechanisms and context must be considered when addressing the dualism between intrinsic or extrinsic cause of patterns.

In fact, Turing's concept of patterning from within had for years faced opposition from the other camp, who maintained that patterns must be pre-laid, typically by gradients of morphogens. Lewis Wolpert [27] championed the idea of positional information in which (in the theoretical simplest case) overlay of two preexisting morphogen gradients oriented in distinct directions would provide a coordinate system, in the form of the relative concentrations of each morphogen at any position, which is then interpreted by the cell and converted into a response.

The vast majority of biologists naturally gravitate toward mechanisms involving external gradients—a convenient and simple notion of causation that does not require comprehension of non-linear dynamics. Obviously, by assuming a preexisting gradient that imposes the asymmetry from outside, one invokes a template and shifts the causation further upstream: Whence the template? What produces the gradient in the first place?

The beauty of the Turing mechanism is that it captures the elementary principle of symmetry-breaking and, thus, epitomizes causation from within, or "self-organization," creating patterns out of a uniform (symmetric) medium without a *deus ex machina*, such as an external gradient. A required ingredient, however, is a configuration of inherent "instability" or "excitability" (embodied by the reaction-diffusion mechanism) of the homogenous medium.

Directional Motility between Extrinsic and Intrinsic Causation

What about the asymmetry of directional motility? In the obvious case of bacteria chemotaxis, extrinsic causation by a preexisting gradient may be an epistemologically satisfying explanation. As to intrinsic causation, the source of the asymmetry can be hard to spot. Symmetry-breaking can arise from intracellular or intercellular mechanisms. The former is most prosaically epitomized by the polarization of an individual cell that generates a front and rear end (Fig 3B) [6,7]. Intercellular dynamics are mediated by signaling molecules that are secreted by the cells themselves and stimulate directional migration, leading to symmetry-breaking at the level of entire cell populations. The formation of a spiral wave pattern arrangement of cells from a uniform spatial distribution in the development of a multicellular aggregate in the amoeba *Dictyostelium discoideum* has become a paradigm for Turing patterns [28,29]. The symmetry-break involves a reaction-diffusion system with the diffusing chemoattractant molecule cAMP that the migrating cells release and also degrade. Here, the individual cell actually sees an externally imposed gradient, generated by the cell population as a whole. Thus, the dualism between external instruction and intrinsic symmetry-breaking can be convoluted.

A new work by Tweedy, Insall, and colleagues [30] on the cooperative directed motility of groups of cells adds a new twist to this dualism of intrinsic and extrinsic cause of asymmetry: the generation of a gradient in an external and uniformly distributed chemoattractant by the cells. Tweedy et al. do not study Turing patterns, although they use *D. discoideum*; instead, they study the persistent directed migration in a chemoattracting nutrient molecule, folate, which is not produced by the cells (in which case it would obviously form a local gradient as in the case of cAMP). They also do not create the scenario of bacterial chemotaxis along an external gradient, because folate is provided uniformly in the dish. There is no "excitable medium" as in the case of Turing patterns [29]. Yet, in this passive and homogeneous medium, the cells collectively generated a local gradient of folate and formed a migrating singleton wave of cells: the cells degraded the nutrient as they moved forward, such that the cells just behind the wave front sense no folate and engage in default random walk [31]. Biochemical measurement of folate in the agarose substrate confirmed the concentration gradient generated by the cells.

This motility fits neither in the category of classical migration up an existing external gradient nor in that of intrinsic pattern formation through a Turing mechanism. But the sustained directional movement in an initially uniform, non-excitable, non-gradient field of a chemoattractant resulting in a "macroscopic pattern" (wave) appears to fit the definition for symmetry-breaking. But where is the symmetry-breaking event exactly?

Even within Externally Imposed Directionality There Is Symmetry-Breaking

Asymmetries induced by instruction from an already asymmetric external cue do not require that an individual cell actually senses the gradient as such. It can, and often does, emanate from a localized symmetry-breaking event at the cellular level, namely in harnessing the undirected random walk to increase persistence in any one direction (see Fig 1). As discussed earlier, overcoming the random turns is the critical step to increase persistence, which is the basis for directional guidance by an external gradient. Bacteria and protozoa are too small to sense

concentration differences across the distance of their cell body size. The net direction instead results from regulated, transient, and timed suppression of random walking by increasing migratory persistence [2,8]. Briefly, the chemoattractant biases the stochastic turns by promoting persistence when the bacteria sense an increase in chemoattractant concentration. In other words, the random direction of persistence, the actual symmetry-break, is subject to selection by the environment, akin to the natural selection of random mutations through which the environment steers the direction of evolution.

Mechanistically, a molecular signaling network relays the signal of receptors bound by the attractant to signal to the flagellar motor, which switches its operation from the mode of tumbling (producing random walk) to the mode of propeller function (increasing persistence) [2,8]. In vertebrate cells, several types of mechanisms have been described that suppress the symmetric random motility and channel it in one dominating direction to promote persistent motion [7]. In a canonical system, the first step consists of cytoskeletal polarization in an initially asymmetric cell, creating a leading and trailing edge of the cell (Fig 3B). Herein lies the symmetry-breaking. The axis of this initial polarization can occur at random but is biased in the presence of a chemoattractant signal that stimulates and stabilizes the formation of the membrane protrusions at the leading edge.

Interestingly, in *D. discoideum*, random walk already has a relatively high baseline persistence [31], similar to some vertebrate cells, such as endothelial cells [12,32,33]. This is achieved by the individual cell remembering the last turn and choosing the direction of the next turn not randomly but with a higher probability being in the opposite direction of the last turn [31]. Persistence appears to be tunable (although this remains to be shown in this case) and, if so, could be exploited to selectively increase persistence when the persistent locomotion by chance points to the direction of the chemoattractant gradient.

In summary, even externally imposed directionality (Fig 1C) depends on symmetry-breaking at a lower, microscopic level, in the intracellular machinery that overcomes the symmetry of random walk (Fig 1D)—although in most cases the detailed molecular mechanism for this process has not been elucidated. But it is in this sense that Ilya Prigogine proposed that symmetry-breaking and the inevitable uncertainty of its direction (direction of persistence) lies at the source of all order in complex systems [34]: it is the substrate on which deterministic extrinsic causation acts by selecting from among the options offered by the symmetry-break that, in the absence of external cues, would be randomly chosen.

Self-Generated Gradients in the Broader Evolutionary Context

In a more encompassing view, order in nature emerges from symmetry-breaking that overcomes the symmetry of randomness and indifferent homogeneity. A system is poised for such an event because of the intrinsic nature in how the system components interact. Evolution by natural selection therefore did not “invent” directional motility but took advantage of an inherent tendency toward symmetry-breaking.

Interestingly, sequence analyses of the genes that encode the signaling pathways of bacterial chemotaxis suggest that the apparatus we now know to control persistence may be much older than the effectors of motility. While both bacteria and archaea achieve motility through flagella, these are analogues but not homologues: their flagellar proteins share no sequence homology [5,35]. However, there is extensive homology between archaea and bacteria in the canonical chemotaxis proteins, including MCP (sensor), CheA (transmitter), CheY (regulator of response), and FliM (the switch of the flagellar motor), as well as CheB and CheR (which control memory resetting) [35]. Thus, while the evolutionary path remains obscure because of the dynamic mosaicism of archaeal genomes, whole genome sequence comparison suggests that control of random walk had been a critical function early on.

The finding by Tweedy et al. [30] now expands the concept of symmetry-breaking in cell motility in an interesting way. Self-generated local gradients are a surprisingly simple mechanism to achieve apparent persistence and, at the same time, a net directional motion. Thus, internal symmetry-breaking to control random walk and selection of direction by an external gradient are collapsed into one step, resulting in a self-propelling directed migration within an initially uniform environment saturated with the nutrient. This simple but resilient and powerful mechanism exploits the fact that an essential nutrient is homogenously in abundance, such that no searching is needed, for which persistence of random walk and chemotaxis have evolved.

So what is the biological function? It is not far-fetched that, at the core, such self-propelled motility, fueled by using up a locally available resource, epitomizes a mechanism analogous to the relentless directionality of grazing herds or forest fires as they move toward areas of uneaten grass or unburned wood and cannot turn back, thereby producing simple spatial patterns (these are favorite models for theorists studying symmetry-breaking). This mode of directional drive more evidently manifests the might of nature's arrow toward a thermodynamic equilibrium. Such self-propelling spatial expansion is much simpler and robust compared to the Turing mechanism, which depends on an excitable (albeit also uniform) medium to create patterns that are (transiently) far away from thermodynamic equilibrium. Whether it is also involved in pattern formation during development or even drives the spread of cancer or necrotizing infections remains to be seen.

References

1. Sackmann E. How actin/myosin crosstalks guide the adhesion, locomotion and polarization of cells. Biochimica et biophysica acta. 2015; 1853(11 Pt B):3132–42. doi: 10.1016/j.bbamcr.2015.06.012 PMID: 26119326.
2. Mitchell JG, Kogure K. Bacterial motility: links to the environment and a driving force for microbial physics. FEMS Microbiol Ecol. 2006; 55(1):3–16. doi: 10.1111/j.1574-6941.2005.00003.x PMID: 16420610.
3. Longo G, Montevil M, Sonnenschein C, Soto AM. In search of principles for a Theory of Organisms. J Biosci. 2015; 40(5):955–68. PMID: 26648040.
4. Codling EA, Plank MJ, Benhamou S. Random walk models in biology. Journal of the Royal Society, Interface / the Royal Society. 2008; 5(25):813–34. doi: 10.1098/rsif.2008.0014 PMID: 18426776; PubMed Central PMCID: PMCPMC2504494.
5. Bardy SL, Ng SY, Jarrell KF. Prokaryotic motility structures. Microbiology. 2003; 149(Pt 2):295–304. doi: 10.1099/mic.0.25948–0 PMID: 12624192.
6. Lauffenburger DA, Horwitz AF. Cell migration: a physically integrated molecular process. Cell. 1996; 84 (3):359–69. PMID: 8608589.
7. Rorth P. Whence directionality: guidance mechanisms in solitary and collective cell migration. Dev Cell. 2011; 20(1):9–18. doi: 10.1016/j.devcel.2010.12.014 PMID: 21238921.
8. Sourjik V, Wingreen NS. Responding to chemical gradients: bacterial chemotaxis. Curr Opin Cell Biol. 2012; 24(2):262–8. doi: 10.1016/j.ceb.2011.11.008 PMID: 22169400; PubMed Central PMCID: PMCPMC3320702.
9. Berg H. Random Walks in Biology. Paperback ed. Princeton: Princeton University Press; 1993.
10. Dunn GA, Brown AF. A unified approach to analysing cell motility. J Cell Sci Suppl. 1987; 8:81–102. PMID: 3503898.
11. Goodwin BC, Kauffman S, Murray JD. Is morphogenesis an intrinsically robust process? J Theor Biol. 1993; 163(1):135–44. PMID: 8412239.
12. Huang S, Brangwynne CP, Parker KK, Ingber DE. Symmetry-breaking in mammalian cell cohort migration during tissue pattern formation: role of random-walk persistence. Cell Motil Cytoskeleton. 2005; 61 (4):201–13. PMID: 15986404.
13. Li R, Bowerman B. Symmetry breaking in biology. Cold Spring Harb Perspect Biol. 2010; 2(3):a003475. doi: 10.1101/cshperspect.a003475 PMID: 20300216; PubMed Central PMCID: PMCPMC2829966.
14. Zhou JX, Aliyu MD, Aurell E, Huang S. Quasi-potential landscape in complex multi-stable systems. Journal of the Royal Society, Interface / the Royal Society. 2012; 9(77):3539–53. Epub 2012/08/31. doi: 10.1098/rsif.2012.0434 PMID: 22933187.

15. Miyazawa S, Okamoto M, Kondo S. Blending of animal colour patterns by hybridization. Nature communications. 2010; 1:66. doi: 10.1038/ncomms1071 PMID: 20842190; PubMed Central PMCID: PMCPMC2982180.

16. Ball P. Forging patterns and making waves from biology to geology: a commentary on Turing (1952) 'The chemical basis of morphogenesis'. Philos Trans R Soc Lond B Biol Sci. 2015; 370(1666). doi: 10.1098/rstb.2014.0218 PMID: 25750229; PubMed Central PMCID: PMCPMC4360114.

17. Kondo S, Miura T. Reaction-diffusion model as a framework for understanding biological pattern formation. Science. 2010; 329(5999):1616–20. doi: 10.1126/science.1179047 PMID: 20929839.

18. Murray JD. Mathematical biology. Second Edition (1993) ed. Berlin Heidelberg New York: Springer-Verlag,; 1989

19. Sheth R, Marcon L, Bastida MF, Junco M, Quintana L, Dahn R, et al. Hox genes regulate digit patterning by controlling the wavelength of a Turing-type mechanism. Science. 2012; 338(6113):1476–80. doi: 10.1126/science.1226804 PMID: 23239739; PubMed Central PMCID: PMCPMC4486416.

20. Newman SA, Frisch HL. Dynamics of skeletal pattern formation in developing chick limb. Science. 1979; 205(4407):662–8. PMID: 462174.

21. Muller P, Rogers KW, Jordan BM, Lee JS, Robson D, Ramanathan S, et al. Differential diffusivity of Nodal and Lefty underlies a reaction-diffusion patterning system. Science. 2012; 336(6082):721–4. doi: 10.1126/science.1221920 PMID: 22499809; PubMed Central PMCID: PMCPMC3525670.

22. Economou AD, Ohazama A, Porntaveetus T, Sharpe PT, Kondo S, Basson MA, et al. Periodic stripe formation by a Turing mechanism operating at growth zones in the mammalian palate. Nat Genet. 2012; 44(3):348–51. doi: 10.1038/ng.1090 PMID: 22344222; PubMed Central PMCID: PMCPMC3303118.

23. Raspopovic J, Marcon L, Russo L, Sharpe J. Modeling digits. Digit patterning is controlled by a Bmp-Sox9-Wnt Turing network modulated by morphogen gradients. Science. 2014; 345(6196):566–70. doi: 10.1126/science.1252960 PMID: 25082703.

24. Meinhardt H. Orientation of chemotactic cells and growth cones: models and mechanisms. J Cell Sci. 1999; 112 (Pt 17):2867–74. PMID: 10444381.

25. Briscoe J, Small S. Morphogen rules: design principles of gradient-mediated embryo patterning. Development. 2015; 142(23):3996–4009. doi: 10.1242/dev.129452 PMID: 26628090; PubMed Central PMCID: PMCPMC4712844.

26. Gavis ER, Lehmann R. Translational regulation of nanos by RNA localization. Nature. 1994; 369 (6478):315–8. doi: 10.1038/369315a0 PMID: 7514276.

27. Wolpert L. Positional information revisited. Development. 1989; 107 Suppl:3–12. PMID: 2699855.

28. Nagano S. Modeling the model organism Dictyostelium discoideum. Dev Growth Differ. 2000; 42 (6):541–50. PMID: 11142676.

29. Hofer T, Sherratt JA, Maini PK. Dictyostelium discoideum: cellular self-organization in an excitable biological medium. Proc Biol Sci. 1995; 259(1356):249–57. doi: 10.1098/rspb.1995.0037 PMID: 7740045.

30. Tweedy L, Knechst D, Mackay G, Insall R. Self-Generated Chemoattractant Gradients: Attractant Depletion Extends the Range and Robustness of Chemotaxis. PLoS Biol. 2016; XXX(XXX).

31. Li L, Norrelykke SF, Cox EC. Persistent cell motion in the absence of external signals: a search strategy for eukaryotic cells. PLoS ONE. 2008; 3(5):e2093. doi: 10.1371/journal.pone.0002093 PMID: 18461173; PubMed Central PMCID: PMCPMC2358978.

32. Ware MF, Wells A, Lauffenburger DA. Epidermal growth factor alters fibroblast migration speed and directional persistence reciprocally and in a matrix-dependent manner. J Cell Sci. 1998; 111 (Pt 16):2423–32. PMID: 9683636.

33. Stokes CL, Lauffenburger DA. Analysis of the roles of microvessel endothelial cell random motility and chemotaxis in angiogenesis. J Theor Biol. 1991; 152(3):377–403. PMID: 1721100.

34. Prigogine I. The End of Certainty 1 ed. New York NY: The Free Press; 1997 August 17, 1997. 240 p.

35. Faguy DM, Jarrell KF. A twisted tale: the origin and evolution of motility and chemotaxis in prokaryotes. Microbiology. 1999; 145 (Pt 2):279–81. doi: 10.1099/13500872-145-2-279 PMID: 10075408.
